# Bibliometric analysis of hypoxia inducible factor prolyl hydroxylase inhibitor in anemia

**DOI:** 10.3389/fphar.2022.1005225

**Published:** 2022-09-21

**Authors:** Li Zheng, Ming Liu, Yatong Zhang, Kaihua Zhang, Yanting Gu, Deping Liu

**Affiliations:** ^1^ Department of Pharmacy, China Aerospace Science & Industry Corporation 731 Hospital, Beijing, China; ^2^ Evidence-Based Medicine Center, School of Basic Medical Sciences, Lanzhou University, Lanzhou, China; ^3^ Department of Pharmacy, Beijing Hospital, National Center of Gerontology, Institute of Geriatric Medicine, Chinese Academy of Medical Sciences, Beijing, China; ^4^ Thoracic Surgery Department, China Aerospace Science & Industry Corporation 731 Hospital, Beijing, China; ^5^ Department of Cardiovascular Medicine, Beijing Hospital, National Center of Gerontology, Institute of Geriatric Medicine, Chinese Academy of Medical Sciences, Beijing, China

**Keywords:** bibliometric analysis, research status, hot topics, hypoxia inducible factor prolyl hydroxylase inhibitor, web of science core collection database

## Abstract

**Objective:** This study aimed to explore the global research status, hot topics, and future prospects in the field of the hypoxia inducible factor prolyl hydroxylase inhibitor (HIF-PHI) by bibliometric analysis.

**Methods:** The literatures about HIF-PHI were downloaded from the Web of Science Core Collection and Pubmed database from inceptions to January.10th. 2022. The VOSviewer 1.6.18 was used to explore the bibliometric networks and research priorities of HIF-PHI.

**Results:** A total of 409 papers about HIF-PHI were included, involving 1,674 authors from 548 institutions in 43 countries. The number of HIF-PHI literatures showed an upward trend, with steady growth from 2016 to 2020 and rapid growth in 2021. Tadao Akizawa, Masaomi Nangaku and Alexander R Cobitz published the most literatures. The United States, Japan and China contributed the most publications. The three most contributed institutions are Astellas Pharma Inc., the Showa University and Glaxosmithkline. Therapeutic Apheresis and Dialysis, American Journal of Nephrology and Clinical Pharmacology in Drug Development are the most productive journals. The main hot topics of HIF-PHI field are anemia, chronic kidney disease, hif-phi, epoetin and roxadustat.

**Conclusion:** The United States and Japan are dominant in the field of HIF-PHI research. The discovery and clinical application of HIF-PHI is a great boon for patients with renal anemia. However, due to the short clinical application time of HIF-PHI, and its long-term efficacy and safety still need time to prove. In addition, more cooperation should be carried out between European and American countries and Asian countries to better prove the clinical value of HIF-PHI.

## Introduction

Renal anaemia is a common complication of chronic kidney disease (CKD) and its incidence increases with the progression of CKD (Zheng et al., 2022). The causes of renal anemia include erythropoietin (EPO) deficiency, iron deficiency, disturbance of iron metabolism in the body and resistance of the EPO signaling pathway (Haase, 2017). The absolute or relative deficiency of EPO is the most important factor leading to anemia. At present, the main treatment for renal anemia is erythropoietin-stimulating agent (ESA) combined with iron, but study has found that ESA may increase the risk of cardiovascular and cerebrovascular events and tumors in patients (Cernaro et al., 2014). In addition, this treatment method also faces some clinical problems, such as patients with EPO resistance, iron utilization disorders, the safety of long-term using intravenous iron, and so on (Babitt and Lin, 2012). It can be seen that the current clinical use of ESA has been limited to a certain extent. With the continuous in-depth exploration of the changes in the body’s cells’ oxygen sensing and adaptive response, the hypoxia-inducible factor prolyl hydroxylase inhibitor (HIF-PHI), a new drug targeting hypoxia-inducible factor prolyl hydroxylase, has become a new way to treat renal anemia, and it is also a kind of oral treatment of renal anemia (Yap et al., 2021). China is the first country in the world to use HIF-PHI to treat renal anemia.

Bibliometrics is a method of quantitatively summarizing multi-dimensional information in a certain field. It uses visualization and network-related technologies to explore the research trend of a certain field, which can help researchers grasp the research status of this field and predict future hotspots in a short period of time (Gao et al., 2018). After searching, we did not find any published bibliometric studies related to the HIF-PHI. In this study, we describe the characteristics of the distribution of HIF-PHI literatures and explore the hot research topics through a bibliometric analysis. This study aims to provide reference and help for researchers, clinicians and patients in this field.

## Methods

### Search strategies

We searched publications about HIF-PHI by using the Web of Science Core Collection (WoSCC) and Pubmed database from inceptions to January.10th. 2022, and develop a complete search strategy with the help of a literature search expert. The search terms that we used in the WoSCC were as follows: TS=(roxadustat OR FG-4592 OR FG 4592 OR FG4592 OR daprodustat OR GSK-1278863 OR GSK 1278863 OR GSK1278863 OR duvroq OR vadadustat OR AKB-6548 OR AKB 6548 OR AKB6548 OR PG-1016548 OR PG 1016548 OR PG1016548 OR B-506 OR B506 OR MT-6548 OR MT6548 OR MT 6548 OR vafseo OR enarodustat OR JTZ-951 OR JTZ951 OR JTZ 951 OR enaroy OR molidustat OR BAY85-3934 OR BAY-85-3934 OR BAY 85-3934 OR BAY 853934 OR BAY853934 OR BAY-853934 OR desidustat OR ZYAN1-1001 OR ZYAN1 OR hypoxia inducible factor prolyl hydroxylase inhibitors OR HIF-PHIs). After the search is complete, the documents were exported in full text format.

### Inclusion criteria

A literatures was included in this study if the following criteria were satisfied: 1) the literature types are “ARTICLE” and “REVIEW”; 2) the research topic is related to HIF-PHI; 3) reports that were written in English. Two researchers (Yatong Zhang and Kaihua Zhang) independently screened the retrieval records according to the inclusion criteria, checked them one by one after completion. In the case of a disagreement, a third reviewer (Ming Liu) was involved in finally reaching a consensus through negotiation.

### Data analysis

We exported the full record of data from WoSCC and Pubmed database, including the number of annual publications; outputs of countries/regions, journals, authors and total citations; impact factor (IF) in 2022 and Hirsch index (H-index). H-index can be used to evaluate the amount and level of academic output of researchers or countries/regions.

Then, we imported the data to Microsoft Excel 2016 and VOS viewer 1.6.18 for further analysis. Microsoft Excel 2016 was used to create a trend chart of changes in the volume of documents issued each year, top 10 journals and numbers of published papers, top 10 scholars and their countries and institutions ranked by the number of publications and citations, citations per article, total number of citations and H-index of top five countries/regions.

We used VOSviewer 1.6.18 to visualize collaborations between countries, institutions and authors and conduct cocitation analysis of authors, journals and references. In addition, we explored the changes in research directions and trends by creating a timeline view of co-cited reference. In order to better explore research hotspots, we used VOSviewer to visualize keywords.

## Results

### Included studies

**FIGURE 1:**
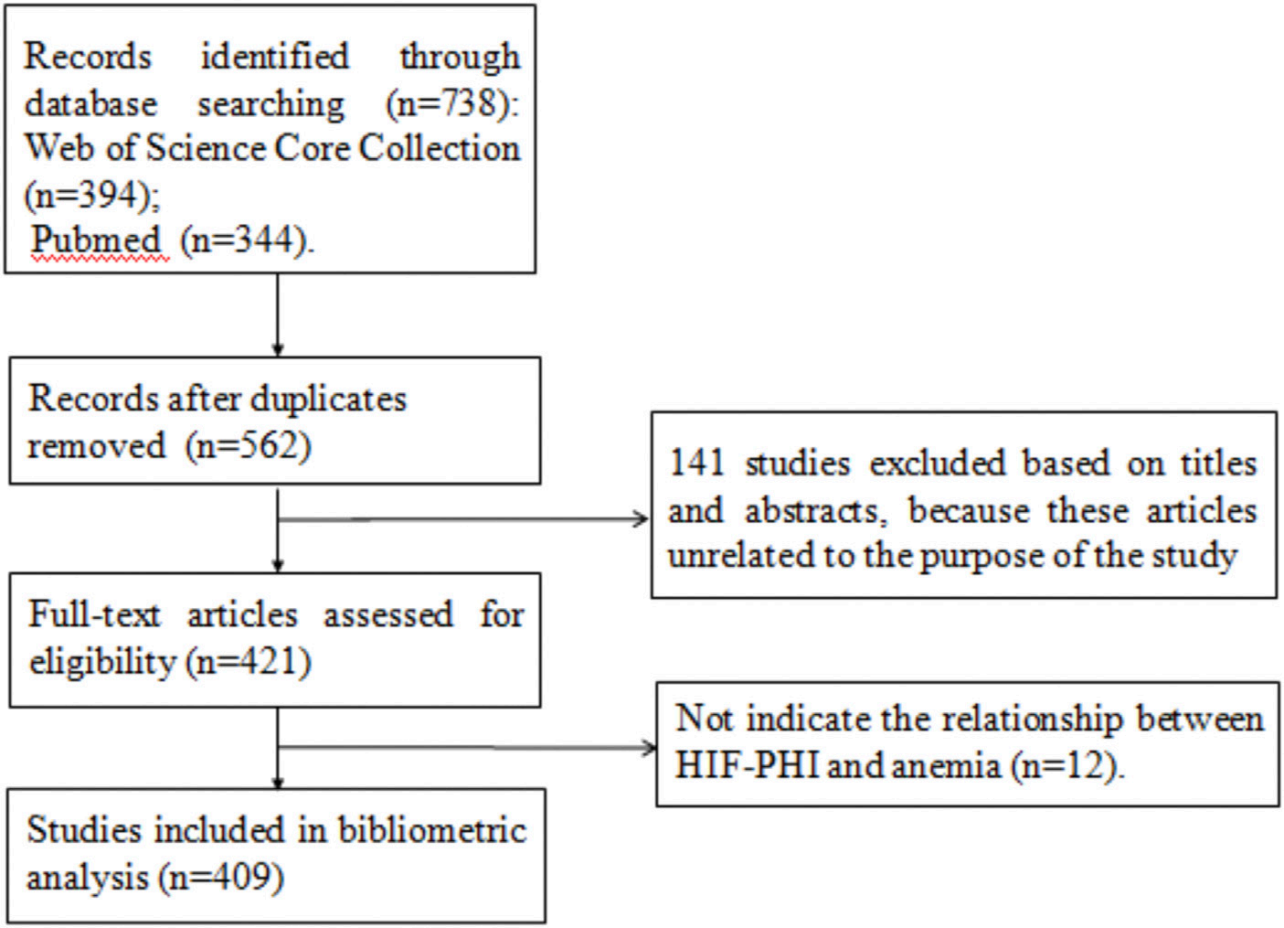
Search process (PRISMA flowchart).

Initially, 738 articles were obtained from the WoSCC and Pubmed database. Of these, we deleted 176 studies by searching for duplicate, 141 articles were excluded based on their titles and abstracts and 12 articles were excluded after reading the full text. 409 articles were finally included. See Figure 1 for the search process.

### Distribution of annual publications

**FIGURE 2:**
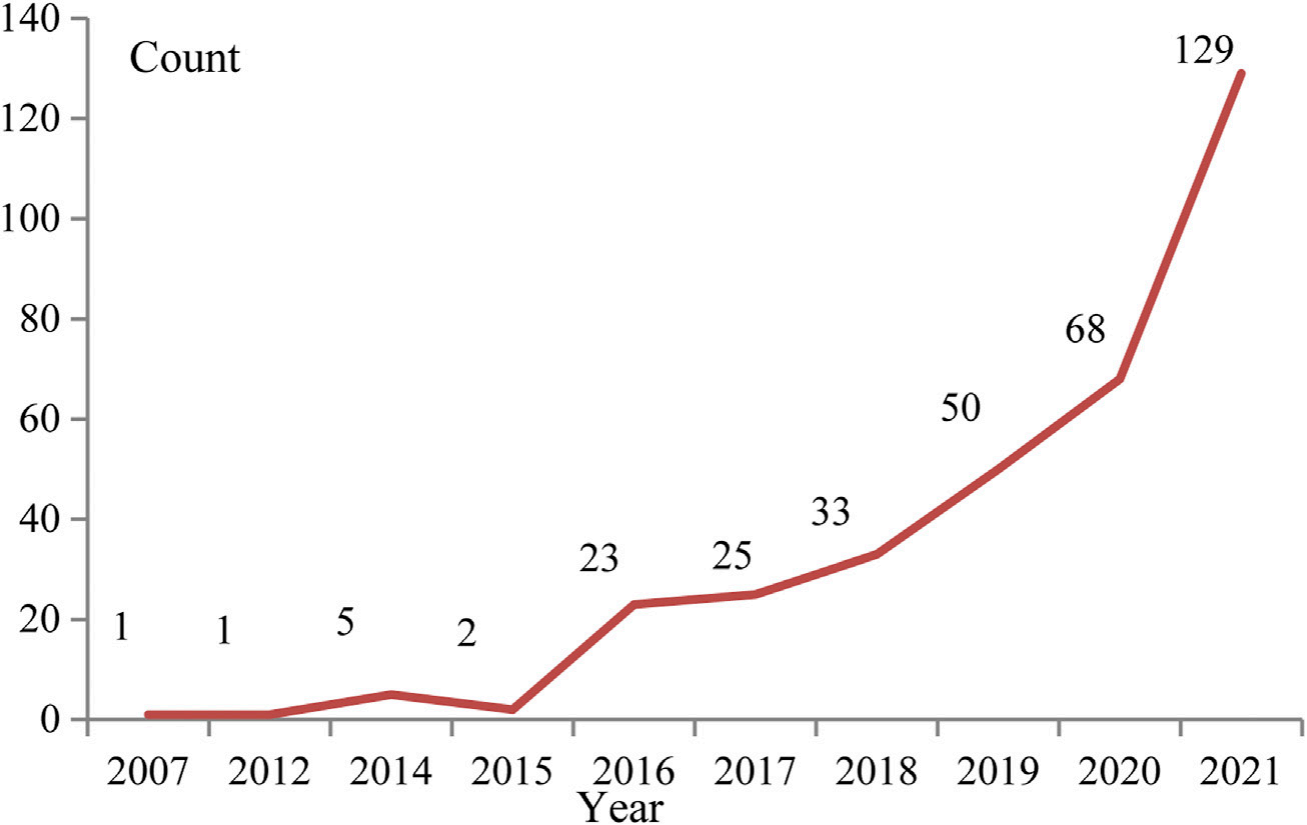
Change chart of annual number of issued papers.

A total of 409 studies were finally included, the average citation from the included studies was 20.22. The earliest 4 studies were published in 2007. Since 2016, the number of annual publications has been more than 10, and the average annual increase of about 10 articles between 2016 and 2020. In 2021, the number of published papers has exceeded 100, reaching 129. Judging from the overall trend of publications, the annual publication volume shows an increasing trend year by year (Figure 2).

### Author analysis

**FIGURE 3:**
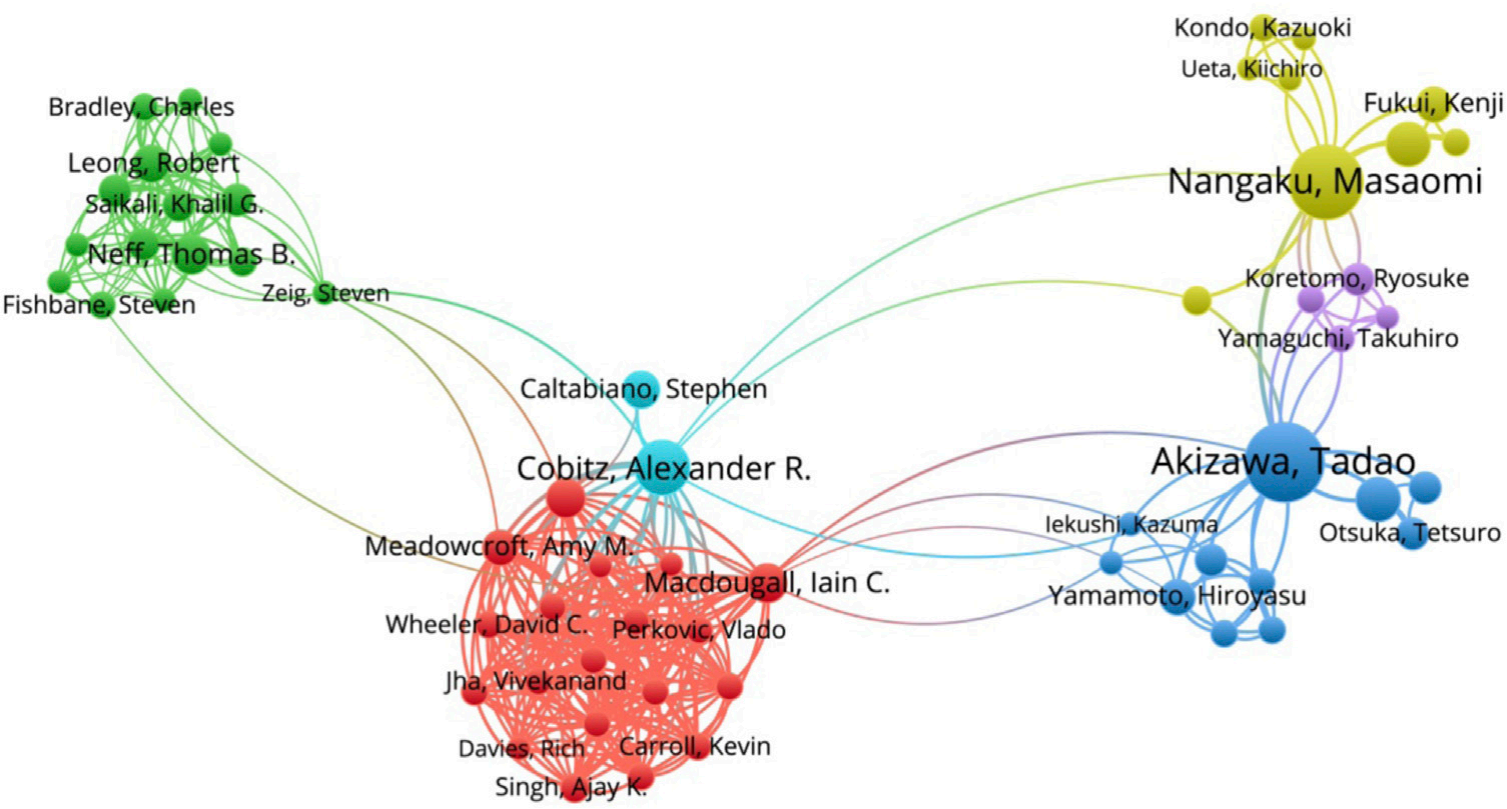
Network map of authors cooperation.

The included 409 publications were produced by 1,674 authors, all authors had collaborative relationships of varying strength, and top 60 authors with association strengths were included for secondary analysis. The results showed that the top 3 scholars for collaborative research in this field are Tadao Akizawa, Masaomi Nangaku and Robert Leong (Figure 3). It can be seen from the Figure 3 that there was a close cooperation relationship between Tadao Akizawa and Masaomi Nangaku from Japan, and there was no cooperation relationship between Robert Leong from the United States and Japanese authors. We can see that these authors cooperate more with domestic scholars than with foreign authors.

Tables 1, 2 list the top 10 scholars and their countries and institutions in terms of the number of papers published and the number of citations. Tadao Akizawa headed with 48 documents, followed closely by Masaomi Nangaku with 36 documents. Thomas B. Neff and Tadao Akizawa were both the most productive and co-cited authors. As we can see from Table 2, the most frequently co-cited author was Thomas B. Neff (887 citations), who is also the fifth most prolific author (n = 18). Among the top 10 authors by both the most published articles and co-cited authors were Tadao Akizawa, Masaomi Nangaku, Thomas B. Neff, Robert Leong, Anatole Besarab and Robert Provenzano.

**TABLE 1:** Nationality and working place of the top 10 high-productive authors.

Rank	Author	Documents	Countries	Institution
1	Tadao Akizawa	48	Japan	Showa University
2	Masaomi Nangaku	36	Japan	The University of Tokyo
3	Alexander R Cobitz	22	UK	GlaxoSmithKline
4	Michael Reusch	20	Netherlands	Astellas Pharma Europe B.V
5	Thomas B. Neff	18	United States	FibroGen Inc.
6	Tetsuhiro Tanaka	17	Japan	The University of Tokyo
7	Robert Leong	16	United States	FibroGen Inc.
8	Hiroyasu Yamamoto	12	Japan	The Jikei University School
9	Anatole Besarab	11	United States	Stanford University School
9	Robert Provenzano	11	United States	Wayne State University School

**TABLE 2:** Nationality and working place of the top 10 co-cited authors.

Rank	Author	Citation	Countries	Institution
1	Thomas B. Neff	887	United States	FibroGen Inc.
2	Anatole Besarab	850	United States	Stanford University School
3	Stefan Hemmerich	816	United States	FibroGen Inc.
4	Robert Leong	624	United States	FibroGen Inc.
5	Glenn M Chertow	613	United States	Stanford University School of Medicine
6	Tadao Akizawa	592	Japan	Showa University
7	Robert Provenzano	586	United States	Wayne State University School
8	Khalil G Saikali	584	United States	FibroGen Inc.
9	Masaomi Nangaku	567	Japan	The University of Tokyo

### Journal analysis

In total, 142 journals have contributed to the 409 publications. Table 3 lists the top 10 journals by publication volume. The statistical results found that the number of studies published in these 10 journals accounted for 22.98% (n = 94) of the total number of studies. Among them, the top 3 journals in terms of publication volume are “Therapeutic Apheresis and Dialysis (n = 13, IF _2022_ = 2.195)”, “American Journal of Nephrology (n = 12, IF _2022_ = 4.605)” and “Clinical Pharmacology in Drug Development (n = 12, IF _2022_ = 2.151)”.

**TABLE 3:** Top10 journals and numbers of published papers.

Rank	Journal	Published papers	IF (2022)
1	Therapeutic Apheresis and Dialysis	13	2.195
2	American Journal of Nephrology	12	4.605
3	Clinical Pharmacology in Drug Development	12	2.151
4	Nephrology Dialysis Transplantation	12	7.186
5	Frontiers in Pharmacology	9	5.988
6	Kidney International Reports	9	6.234
7	Current Opinion in Nephrology and Hypertension	8	3.416
8	Journal of the American Society of Nephrology	7	14.978
9	New England Journal of Medicine	6	176.079
10	Kidney International	6	18.998

### Country and institution analysis

All publications in the field were distributed among 548 institutions from 43 countries, and the strength of the partnership varies between countries and between institutions. Among them, 17 countries and 41 institusions participated in publishing no less than 5 articles. To explore country and institution cooperation, we constructed a network visualization map for publications on HIF-PHI by VOSviewer. The results showed that the top three collaborations countries were the United States, Germany and the UK (Supplementary Figure S1), and the top three collaborations institutions were GlaxoSmithKline, Showa University, The University of Tokyo and Kings College London (Supplementary Figure S2).

**FIGURE 4:**
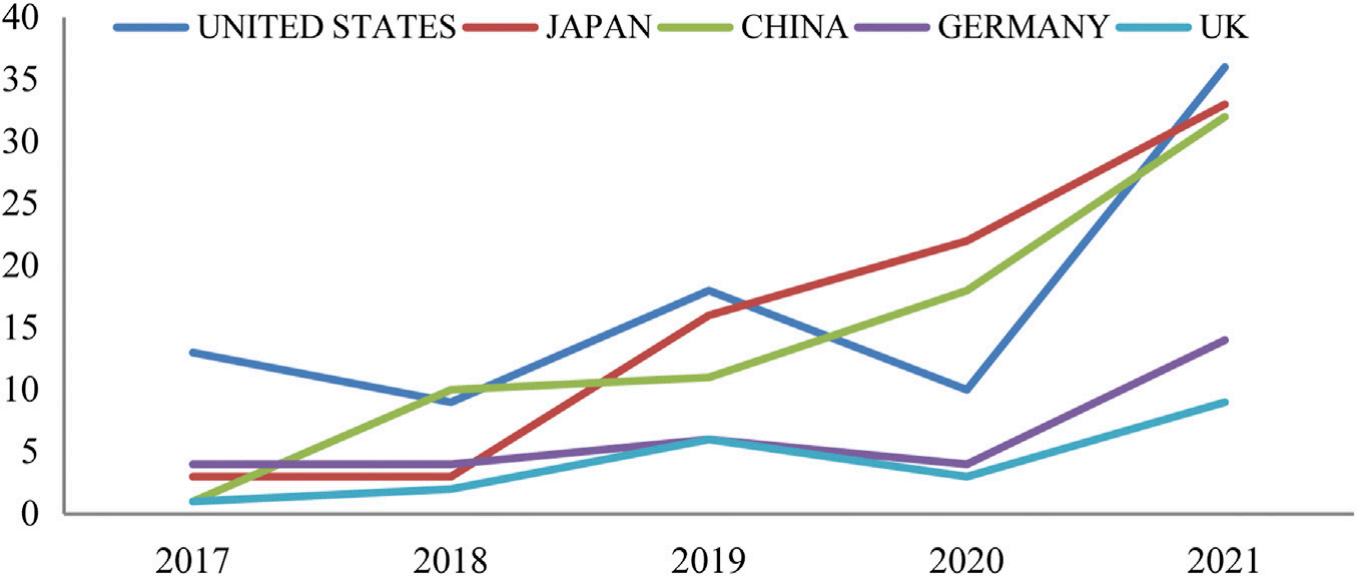
The annual HIF-PHI research output of the most 5 high-productive countries.

The top 5 countries by the number of published articles are the United States, China, Japan, Germany and UK. In the past 5 years, the annual research volume in China has shown an obvious increasing trend (Figure 4). Among the H-index rankings of research in different countries, the United States has the highest H-index, the highest total citations and the highest average citations, and the UK has the lowest H-index among the five countries, but it ranks second in median of citations. The total citations, average citations and H-index comparative analysis results of HIF-PHI related research in the top 5 countries with published papers were shown in the Table 4.

**TABLE 4:** Citation (all, median) and H-index of hif-phi research in top 5 countries.

No.	Countries	H-index	Total of citation	Median of citation
1	United States	33	3,992	35.96
2	Japan	21	1,146	13.33
3	China	18	1,577	17.52
4	Germany	18	823	20.07
5	UK	16	674	25.92

The institution with the highest number of publications was Astellas Pharma Inc., followed closely by Showa University. Table 5 showed the information results of the top 10 institutions with the most published papers. Among the top 10 institutions, 5 institutions were from Japan, including universities, hospitals and biotechnology companies. In addition, 5 of the top 10 institutions were biotechnology companies, indicating that most of the related research on HIF-PHI focuses on biotechnology companies.

**TABLE 5:** Number of publications and countries of the top 10 institutions.

No.	Institutions	Countries	Number of publications (%)
1	Astellas Pharma Inc.	Japan	34 (8.31)
2	Showa University	Japan	31 (7.58)
3	GlaxoSmithKline	UK	30 (7.33)
4	The University of Tokyo	Japan	28 (6.85)
5	Japan Tobacco Inc.	Japan	18 (4.40)
6	FibroGen Inc.	United States	15 (3.67)
7	Bayer Yakuhin	Germany	12 (2.93)
8	Kings College London	UK	11 (2.69)
9	Vanderbilt University	United States	10 (2.44)
10	Nanjing Medicine University	China	9 (2.20)

### Keyword analysis

**FIGURE 5:**
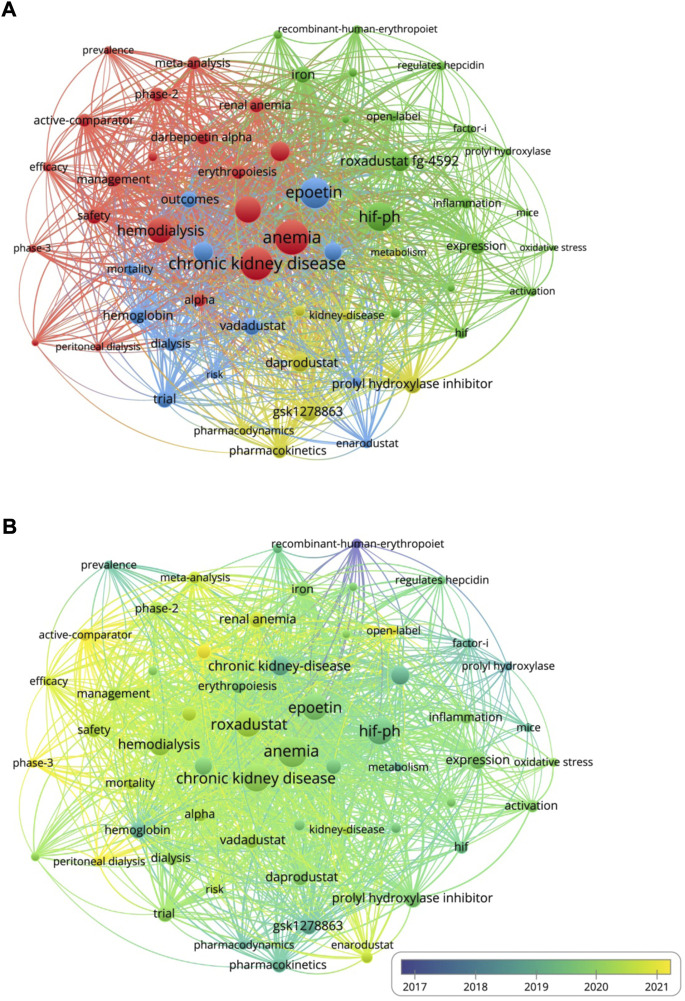
**(A)** Network map of cooccurrence keywords. **(B)** The year-based overlay visualization of the keywords.

A total of 1,131 keywords were finally included, and the top 5 keywords with co-occurrence were anemia (n = 169), chronic kidney disease (n = 139), hif-phi (n = 131), epoetin (n = 149) and roxadustat (n = 114). We used VOSviewer (Figure 5A) to analyze the keywords and formed four clusters: the red cluster research topic is related to the treatment of renal anemia with roxadustat, the green cluster research topic is related to the basic research related to HIF-PHI, the blue cluster research topic is related to the treatment of anemia with epoetin, and the yellow cluster research topic is mainly related to the clinical application of HIF-PHI.

We used VOSviewer to color-code the time that different keywords appear in their corresponding literature, and generate a trend graph of HIF-PHI research directions over time (Figure 5B). It can be seen from the Figure 5B that the early research mainly focused on the treatment of recombinant human erythropoiet (rhEPO) for anemia, and gradually developed to drug treatment and clinical research over time (blue). Yellow-green colors represent the latest research topics, and the representative keywords were roxadusat, vadadustat, daprodustat and anemia. The results show that roxadusat, vadadustat and daprodustat have received more attention in recent years.

### Cited reference analysis

A total of 6,835 papers were cited in the included papers, and 177 papers were cited more than 10 times. The top 3 papers with the most citations were: “Besarab A, 2015, Nephrol Dial Transpl, V30, P1665 (n = 109)”, “Singh AK, 2006, New Engl J Med, V355, P2085 (n = 99)” and “Pfeffer ma, 2009, New Engl J Med, V361, P2019 (n = 98)”. The related information of the top 10 papers with the most citations was shown in Table 6. Only one of the 10 papers is a review article, and the others are clinical trials.

**TABLE 6:** Top 10 co-cited references in HIF-PHI.

Rank	Co-cited reference	Number of citations	Article type
1	Besarab A, 2015, Nephrol Dial Transpl, v30, p1665	109	Clinical Trial
2	Singhak, 2006, New Engl J Med, v355, p2085	99	Controlled Trial
3	Pfeffer Ma, 2009, New Engl J Med, v361, p2019	98	Controlled Trial
4	Provenzano R, 2016, Am J Kidney Dis, v67, p912	98	Controlled Trial
5	Chen N, 2019, New Engl J Med, v381, p1011	95	Controlled Trial
6	Chen N, 2019, New Engl J Med, v381, p1001	89	Controlled Trial
7	Provenzano R, 2016, Clin J Am Soc Nephrol, v11, p982	88	Controlled Trial
8	Besarab A, 2016, J Am Soc Nephrol, v27, p1225	80	Controlled Trial
9	Gupta N, 2017, Am J Kidney Dis, v69, p815	75	Review
10	Pergola Pe, 2016, Kidney Int, v90, p1115	74	Controlled Trial

**FIGURE 6:**
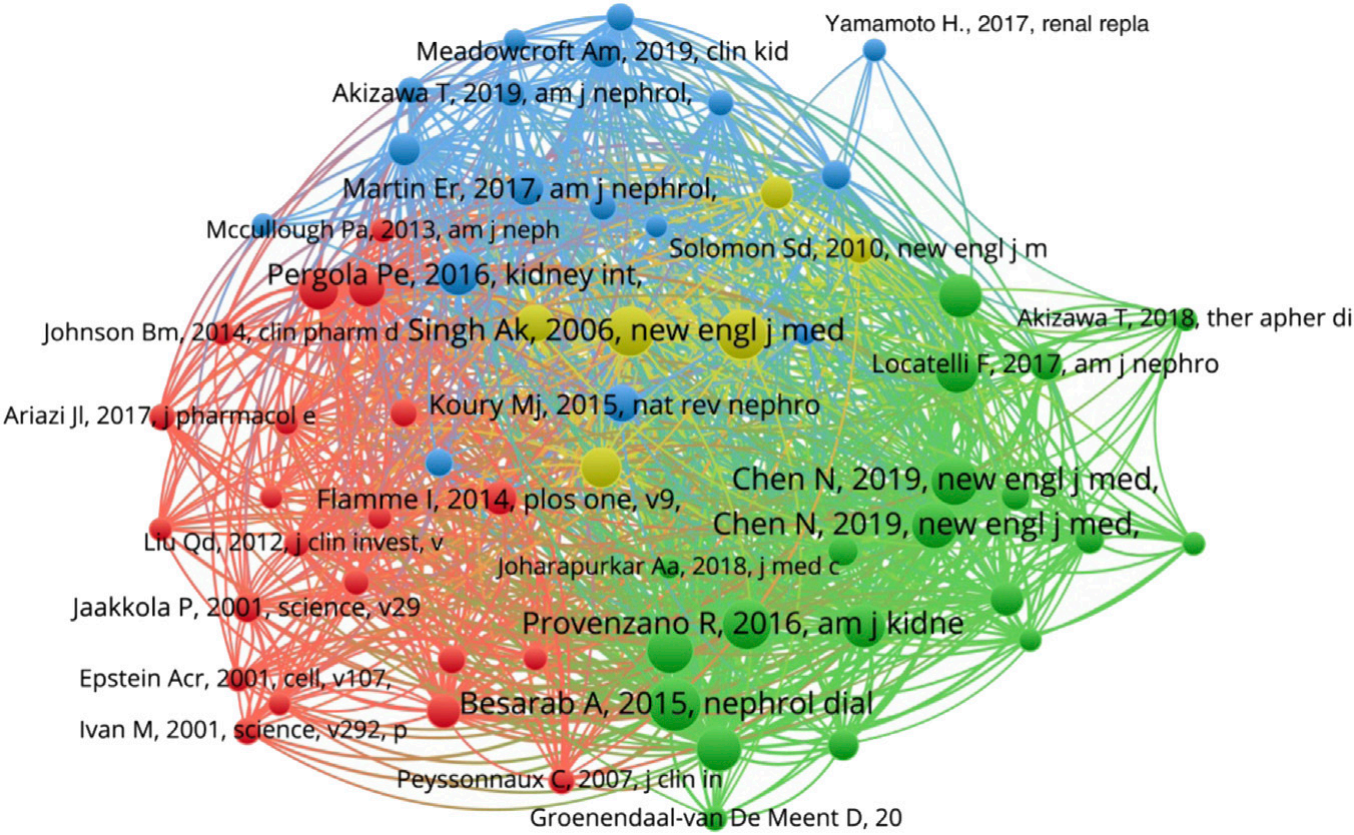
The co-word network visualization of co-cited references in HIF-PHI.

Figure 6 present the co-citations of references, the largest nodes represent the most cited times and strong betweenness centrality. The document with the largest node was Besarab A, 2015, Nephrol Dial Transpl, V30, P1665 (n = 109), indicating that this document had the most citations.

## Discussion

According to the analysis of annual publications volume, the number of published studies on HIF-PHI was based on 2016. Few studies were published before 2016, suggesting that the field has not received enough attention. After 2016, the total number of global studies on HIF-PHI has been increasing year by year, indicating that this field has gained more and more attention. The number of published papers, H-index, total citations and average citations in the United States were all in the world’s leading position, indicating that the United States has contributed the most in the field of HIF-PHI research. China ranked second in the number of published papers, and the number of published papers has shown a significant increase in the past 5 years. In terms of indicators such as total citation and H-index, which reflect academic influence and publication quality, it ranked second and third in the world respectively, indicating that China’s Research in this field has made great progress and has a high influence all over the world. In addition, Europe and the United States pay attention to cooperation with each other, the partnership between the US, Germany and the UK was particularly strong. The cooperation between Japan and Europe and the United States is stronger than that between China and Europe and the United States.

In the field of HIF research, the top 10 journals in terms of publication volume accounted for 22.98% of the total publication volume, journals with more than 5 papers only accounted for 11.27%, and more than 50% of journals published only 1 literature. It shows that the HIF-PHI literature is published in a few journals, but it is widely distributed and intersects with other disciplines. The average impact factor of the top 10 journals is 24.183, of which the New England Journal of Medicine (IF _2022_ = 170.079) has an impact factor of more than 150 and Kidney International (IF _2022_ = 18.998) has an impact factor of more than 15. It shows that the literatures of HIF-PHI research have high quality and the field has received strong attention. In addition, Therapeutic Apheresis and Dialysis, American Journal of Nephrology, Clinical Pharmacology in Drug Development, Nephrology Dialysis Transplantation and Frontiers in Pharmacology are the journals that receive the most HIF-PHI research. Contributors and scholars who track the progress of HIF-PHI research can focus on these journals.

In terms of author analysis, the top three authors with strong co-authorship relationships are Tadao Akizawa, Masaomi Nangaku, and Robert Leong, and these three scholars have a total number of published articles and the H-index of the articles (H index is 14, 15, 10, respectively) also ranked in the top 5 in this field, indicating their outstanding achievements in the field of HIF-PHI research. Among the authors in the included articles, the top 10 authors published more than 18 papers per capita, accounting for 46.24% of the total. They came from Europe, America and Japan and focused on HIF-PHI research. They have achieved fruitful results and have an important position in this field. Among the top 10 procuctive co-cited authors, the top 5 are all from the United States, of which the Thomas B. Neff, Stefan Hemmerich, and Robert Leong are from FibroGen Inc., indicating that FibroGen Inc. made outstanding contributions in this field. 81.96% of the authors only published one article, indicating that there are more researchers involved in HIF-PHI, but fewer researchers focus on this field. An analysis of the topics published by the top three authors found that Tadao Akizawa (Department of Nephrology, Showa University School of Medicine) is mainly engaged in HIF-PHI research (Akizawa et al., 2020a; Akizawa et al., 2020b), Masaomi Nangaku (Tokyo University Graduate School of Medicine) is mainly engaged in HIF-PHI and renal anemia research (Nangaku et al., 2021a; Nangaku et al., 2021b), Alexander R Cobitz works at GlaxoSmithKline, mainly engaged in the research of metabolic pathways and cardiovascular therapy. They are both engaged in research on the efficacy and safety of HIF-PHI in the treatment of renal anemia and are ideal candidates for collaboration in research in this area.

In bibliometrics, frequently occurring keywords can help to discover current research topics and hotspots, and have a certain guiding effect on the development of scientific research (Qiu et al., 2018). The co-occurrence analysis results of VOSviewer showed that the top 5 keywords appeared more than 100 times, namely anemia, chronic kidney disease, hif-phi, epoetin, roxadustat, which were divided into clinical researches, drug treatment research and basic research. These all represent the development trend and hotspot of research. Clinical researches are marked inred and blue, mainly including population studies, suggesting that the focus of the clinical is mainly on data studies based on patients. In terms of drug treatment, the research mainly focuses on the treatment of epoetin, roxadustat and hif-phi for renal anemia. Long-term use of rhEPO in the treatment of renal anemia, some patients are prone to EPO resistance, which may be related to the low response to rhEPO in the body caused by CKD-related inflammatory response and oxidative stress (Locatelli et al., 2006). In recent years, with the deepening of the related research on the changes of the body’s cells sensing oxygen and the adaptive response, the new drug HIF-PHI targeting the hypoxia-inducible factor prolyl hydroxylase has become a new way to treat renal anemia. As the first HIF-PHI developed, roxadustat was the first to complete Phase 3 clinical trials in China and was approved for marketing for the treatment of anemia caused by dialysis-dependent and non-dialysis-dependent chronic kidney disease. The mechanism of action on anemia is mainly to promote the synthesis of EPO (Gerl et al., 2015) and improve iron metabolism (Akizawa et al., 2017; Chen et al., 2017), and it is also beneficial to the cardiovascular aspects of CKD patients (Holdstock et al., 2016; Provenzano et al., 2016). When applying HIF-PHI such as roxadustat to treat renal anemia, it should be noted that ESA only regulates the EPO pathway to promote erythropoiesis, while HIF-PHI acts on a variety of cells and regulates the expression of multiple target genes (Koury and Haase, 2015), including EPO genes and a series of genes related to erythropoiesis, but also genes such as vascular endothelial growth factor that are not related to erythropoiesis but have other functions. Therefore, HIF-PHI may bring benefits that ESA does not have, but its safety issues should also be paid attention to clinically, for example, taking roxadustat may cause hypertension, hyperkalemia and metabolic acidosis (Chen et al., 2019; Shutov et al., 2021). At the same time, it is also necessary to carefully observe the safety problems caused by long-term use of PHI-HIF. At present, the studies on HIF-PHI are phase 2 and 3 clinical studies, and the number of patients included is limited. Now more researchers are exploring the long-term safety of HIF-PHI through real-world studies.

Cited References Analysis showed that a study by Besarab A (FibroGen Inc. United States.) was the most cited, which was published on Nephrology Dialysis Transplantation (IF _2022_ = 7.186) in 2015, and this study is about the efficacy and safety of roxadustat in the treatment of anemia in non-dialysis dependent chronic kidney disease patients (Besarab et al., 2015). Especially in 2021, this study was cited 42 times, indicating that the use of roxadustat for the treatment of renal anemia in patients with non-dialysis chronic kidney disease has received a lot of attention recently. In addition, the second and third most cited papers were written and published by the research teams of Singh AK and Pfeffer Ma, respectively. Both of which were studying the efficacy of traditional treatment regimens on renal anemia in patients with chronic kidney disease (Singh et al., 2006; Pfeffer et al., 2009). This also shows that both HIF-PHI and renal anemia are attracting the attention of researchers.

HIF is a protein complex that can respond to hypoxia and regulate the transcription of specific genes. Gregg Semenza (John Hopkins School of Medicine), William G. Kaelin Jr (Harvard University) and Peter J. Ratcliffe (Oxford University) received the 2019 Nobel Prize in Physiology or Medicine for discovering how the body’s cells sense and react to low oxygen levels through VHL-HIF-PHD mechanism. The work of the three winners proved that there is a direct coupling relationship between the gene expression response triggered by the change of oxygen level and the oxygen level in animal cells, thus allowing the rapid cell level response to the oxidation reaction through the action of HIF transcription factors. This groundbreaking discovery revealed the mechanism of the most important adaptive process in life and laid the foundation for understanding how oxygen levels affect cellular metabolism and physiological functions. The three scientists first discovered and fully expounded the relevant mechanism of HIF signaling pathway in the body’s response to changes in oxygen concentration, and opened up new therapeutic targets for anemia, cardiovascular diseases, macular degeneration, tumors and other diseases.

HIF is mainly regulated by different oxygen states α subunits and constitutively expressed β subunit composition. There are mainly three kinds of α subunits in the body: HIF⁃1α, HIF⁃2α and HIF⁃3α, respectively. HIF⁃1α and HIF⁃2α all mediate the transcriptional response in hypoxia, but their distribution, transcriptional target and oxygen dependence are different (Schödel and Ratcliffe, 2019). Under mild hypoxia, HIF⁃2α can be induced to a greater extent and maintain the active state for a long time, HIF⁃1α needs to be induced to a greater extent under severe hypoxia and its activity peak occurs within the first 24 h of hypoxia (Holmquist-Mengelbier et al., 2006).

The prolyl hydroxylase domain (PHD) in the HIF-α subunit is an important domain that senses oxygen concentration signals *in vivo* and is regulated by PHD hydroxylase. There are three homologous forms of PHD hydroxylase in human body (PHDase 1/2/3) (Bruick and McKnight, 2001). Among them, PHDase2 is the most widely expressed, and in most types of cells, it is the main regulator of HIF-1. The distribution of PHDase1 and PHDase3 is more tissue-specific, and the specific regulation of HIF⁃2 is stronger (Appelhoff et al., 2004). At present, PHIs mostly inhibit three PHDases at the same time.

At present, the HIF-PHI approved for marketing clinically include roxadustat, vadadustat and daprodustat. For the newly approved vadadustat and daprodustat in 2020, the phase III clinical trails had achieved positive effects. The results of PRO2TECT trail and INNO2VATE trial all showed that vadadustat could reach the primary efficacy endpoint (Chertow et al., 2021; Eckardt et al., 2021). However, INNO2VATE trial also confirmed that the primary safety endpoint of vadadustat for non-dialysis patients (NDD-CKD) did not meet expectations, and the heart safety risk still exists. Daprodustat has become the first HIF-PHI that is expected to successfully treat renal anemia, and it does not need to consider whether the patient is in dialysis state. The FDA refused roxadustat to be listed in the United States because it showed some heart related safety problems. The research and development of vadadustat has also suffered setbacks because it may cause the risk related to the heart of NDD-CKD patients. In contrast, the ASCEND-ND trial (Singh et al., 2021) results showed that daprodustat met the predetermined non inferiority criteria for cardiovascular safety compared with darbepoetin alfa.

This paper analyzes related research on HIF-PHI from countries, institutions, journals, authors, references, keywords, etc, and provides the development status and hotspots of HIF-PHI research for scholars interested in this field. However, there are still some limitations in this paper: 1) among the included literatures, some literatures have relatively few citations due to their relatively short publication time, which may bring a certain bias to the co-occurrence analysis results, 2) we verify the publications of the top ten authors carefully to minimize the bias in the analysis due to the same name, but we still cannot avoid the bias caused by the author’s change of institution. 3) since some antuors have the same short name, some keywords have different expressions, although we have standaradized them before analysis, such errors can only be reduced not completely eliminated.

## Conclusion

The United States and Japan are in a leading position in the field of HIF-PHI research. The discovery and clinical application of HIF-PHI is a great boon for patients with renal anemia. However, due to the short clinical application time of HIF-PHI, and its long-term efficacy and safety still need time to prove. In addition, more cooperation should be carried out between European and American countries and Asian countries to better prove the clinical value of HIF-PHI.

## Data Availability

The original contributions presented in the study are included in the article/Supplementary Material, further inquiries can be directed to the corresponding authors.
