# Supplementary figures and images for: Bibliometric analysis of hypoxia inducible factor prolyl hydroxylase inhibitor in anemia
Source: Front Pharmacol. 2022 Sep 21;13:1005225. doi: 10.3389/fphar.2022.1005225 (PMC9549679; doi:10.3389/fphar.2022.1005225)

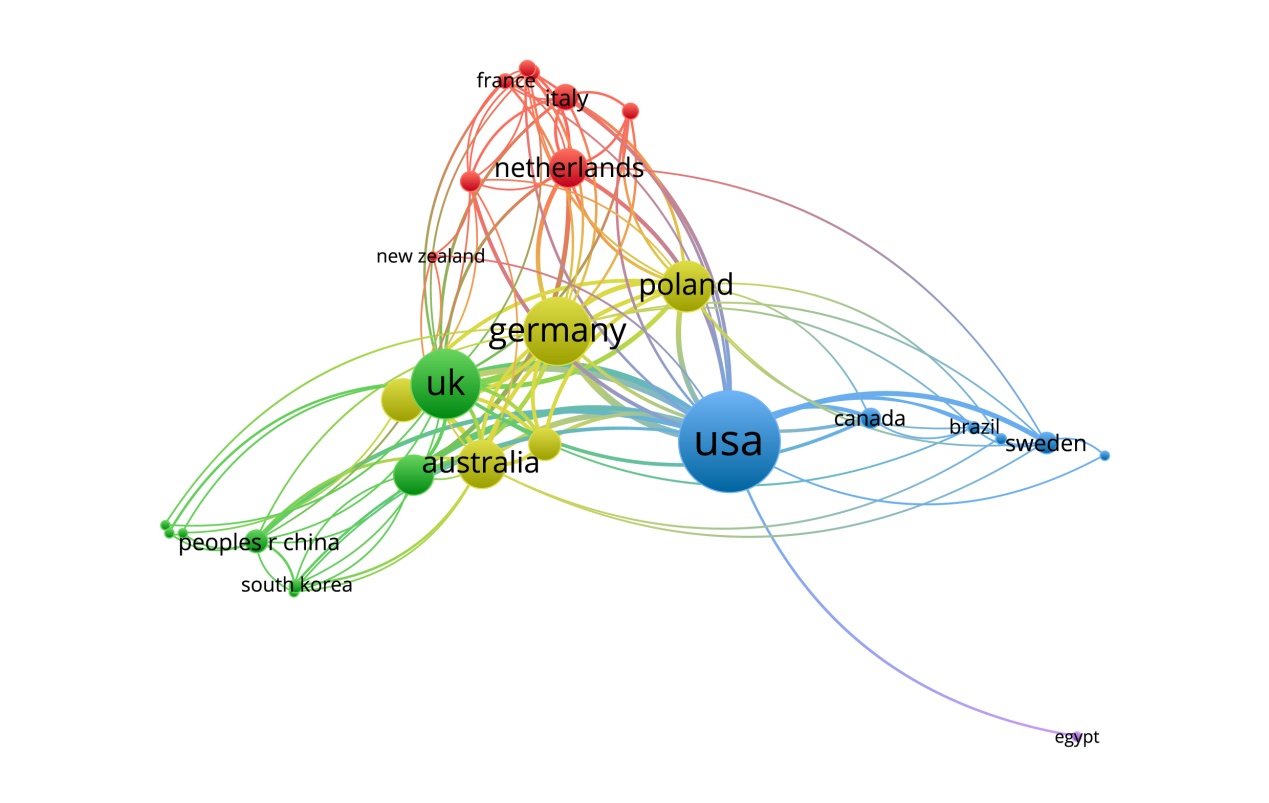


Fig S1. Network map of countries


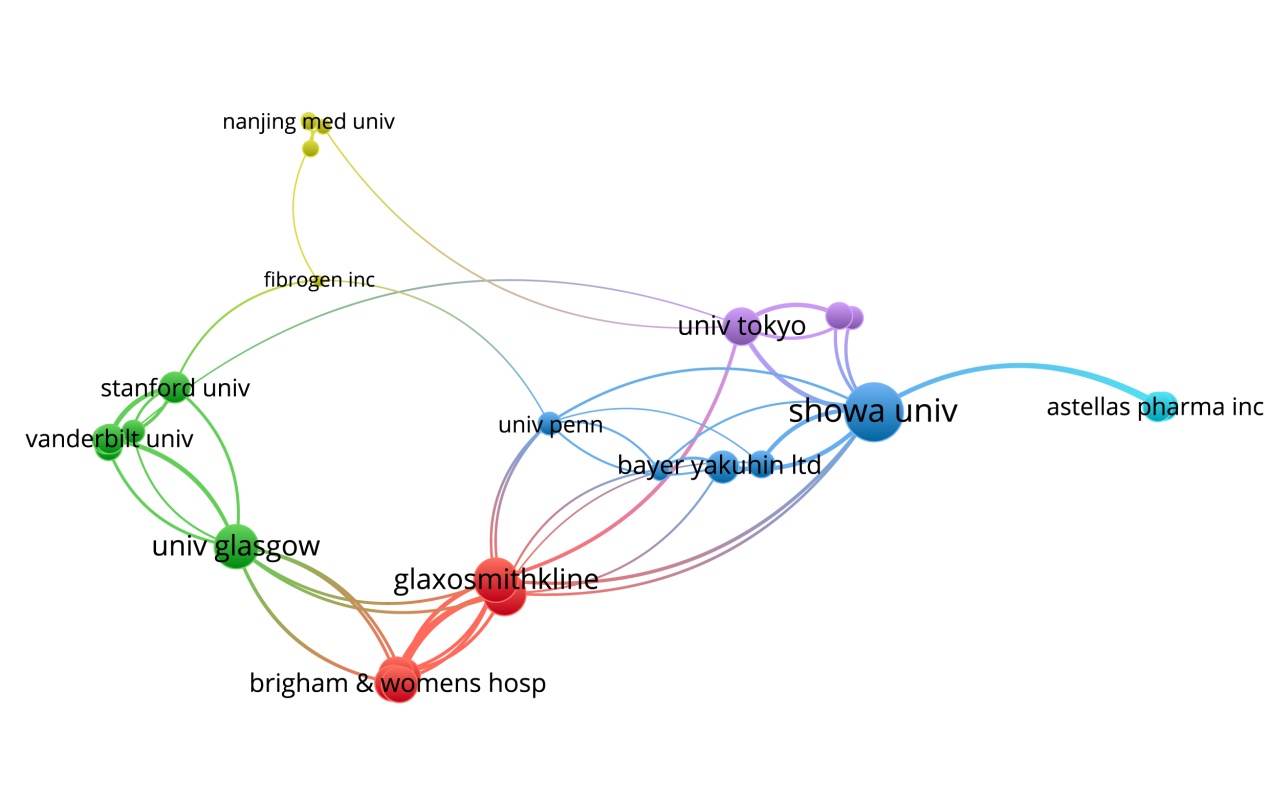


Fig S2. Network map of institutions

Supplement: Supplementary file 1 [file DataSheet1.docx]
